# Identification of pyroptosis subtypes and prognosis model of hepatocellular carcinoma based on pyroptosis‐related genes

**DOI:** 10.1002/cam4.70081

**Published:** 2024-08-09

**Authors:** Haisheng Xie, Guanlin Huang, Haoming Mai, Jiaxuan Chen, Rong Na, De‐Ke Jiang

**Affiliations:** ^1^ State Key Laboratory of Organ Failure Research, MOE Key Laboratory of Infectious Diseases Research in South China, Guangdong Provincial Key Laboratory of Viral Hepatitis Research, Guangdong Provincial Clinical Research Center for Viral Hepatitis, Guangdong Institute of Liver Diseases Department of Infectious Diseases and Hepatology Unit, Nanfang Hospital Southern Medical University Guangzhou China; ^2^ The Key Laboratory of Molecular Pathology (Hepatic Diseases) of Guangxi, Department of Pathology The Affiliated Hospital of Youjiang Medical University for Nationalities Baise Guangxi China; ^3^ Division of Urology, Department of Surgery, LKS Faculty of Medicine The University of Hong Kong Hong Kong China

**Keywords:** hepatocellular carcinoma, multiomic analysis, prognosis model, pyroptosis subtype

## Abstract

**Background:**

Hepatocellular carcinoma (HCC) is a common malignant tumor with poor prognosis. Pyroptosis, a type of programmed cell death, regulates tumor cell development. However, the role of pyroptosis‐related genes (PRGs) in HCC and their association with prognosis are unclear.

**Methods:**

We conducted bioinformatics analysis to identify PRGs in The Cancer Genome Atlas‐Liver Hepatocellular Carcinoma (TCGA‐LIHC) patients. Consensus clustering classified patients into different subtypes. We used LASSO regression to established a pyroptosis subtype‐related score (PSRS) related to prognosis. OncoPredict identified potential pharmaceuticals based on PSRS.

**Results:**

We found 20 HCC‐related PRGs in 335 TCGA‐LIHC patients. Consensus clustering classified patients into two subtypes. Subtype I had better overall survival and higher response to anti‐PD1 treatment. The prognostic model involving 20 genes predicted poorer prognosis for high‐PSRS group. The model was validated in two external cohorts. OncoPredict identified 65 potential pharmaceuticals based on PSRS.

**Conclusion:**

Our investigation revealed a correlation between pyroptosis and HCC. We established PSRS as independent risk factors for predicting prognosis. The study paves the way for using PRGs as prognostic biomarkers and exploring personalized therapy for HCC.

## 1 INTRODUCTION

Hepatocellular carcinoma (HCC) is the primary form of liver cancer, the sixth most common malignancies and the fourth leading cause of cancer‐related deaths worldwide.^1^ HCC typically develops after prolonged periods of chronic hepatitis, liver fibrosis, and cirrhosis.^2^ HCC has extreme heterogeneity and poor prognosis,^3^ with a 5‐year survival rate of only 18%.^4^, ^5^ The current prognosis of HCC relies mainly on clinicopathological staging frameworks, yet several marker characteristics, including *TP53* gene mutations and genes associated with cellular proliferation, have been identified as survival predictors.^6^, ^7^, ^8^ Therefore, there is an urgent need for novel prognostic biomarkers to predict survival and design personalized treatment strategies for patients with HCC.

Pyroptosis is a type of cell death mediated by gasdermins and inflammasomes, which occurs in vertebrates as an innate immune response mechanism.^9^, ^10^ Dysregulation of pyroptosis may lead to impaired pathogen clearance efficacy, dysfunctional adaptive immune response, and tissue damage. Evidence suggests that pyroptosis is involved in the onset and progression of various human diseases, including malignant tumors. Recent studies have shown that chemically‐induced pyroptosis of tumor cells can occur in vitro and in vivo.^11^ As pyroptosis may potentially promote cancer progression by triggering the release of multiple inflammatory mediators, including IL‐1 and IL‐18, some researchers believe that it is a protumorigenic mechanism.^12^, ^13^, ^14^ Nevertheless, recent researches have validated that externally induced pyroptosis exhibits robust antitumor activity.^15^, ^16^

In this study, we investigated the mRNA expression of pyroptosis‐related genes (PRGs) in 355 patients with HCC from The Cancer Genome Atlas (TCGA)‐Liver Hepatocellular Carcinoma (LIHC) database and classified them into distinct pyroptosis subtypes. We further elucidated the relationship between pyroptosis subtypes and prognosis, and developed a prognostic model comprising 20 pyroptosis subtype‐related genes. The model's accuracy was verified in two external HCC cohorts, demonstrating its potential for clinical decision‐making in HCC patient management. Furthermore, we used integrated analysis to scrutinize differences in genomic variations, tumor microenvironment, and immunogenomic patterns between two pyroptosis subtypes. We also identified a novel group of potential drug molecules for patients with HCC based on PSRS, which may provide more therapeutic options. Our study seeks to personalize survival prediction and treatment options for patients with HCC through novel pyroptosis‐based molecular classification and prognostic model.

## 2 MATERIALS AND METHODS

### 2.1 Acquisition of multiomic data of patients with HCC

The current study utilized multiomic data from three HCC cohorts, namely TCGA‐LIHC,^17^ LIRI‐JP,^18^ and CHCC‐HBV.^19^ All transcriptome data were obtained through RNA sequencing, while genomic variations data were obtained through whole genome sequencing. TCGA‐LIHC cohort data were acquired from the Genomic Data Commons (GDC) website (https://portal.gdc.cancer.gov/, accessed on 12 December 2022). LIRI‐JP cohort data were obtained from the International Cancer Genome Consortium (ICGC) portal (https://dcc.icgc.org/projects/LIRI‐JP, accessed on 27 March 2019). CHCC‐HBV cohort data were acquired from The National Omics Data Encyclopedia (NODE) website (https://www.biosino.org/node, accessed on 20 May 2021), and clinical data was obtained from the reference.^19^ Finally, after excluding patients with incomplete clinical information, 335 patients were obtained from TCGA‐LIHC, 233 patients from LIRI‐JP, and 159 patients from CHCC‐HBV.

### 2.2 PRGs selection

PRGs were selected from various sources. First, a group of PRGs were extracted from relevant literatures.^16^, ^20^, ^21^, ^22^, ^23^ Second, the Gene Ontology (GO) database (http://geneontology.org/) was also applied to obtain PRGs, using the keyword “pyroptosis.” Third, the Molecular Signature Database v7.4 (MSigDB) was utilized to download an additional PRGs (Reactome pyroptosis). All PRGs from the above three sources included for further study are summarized in Table S1.

### 2.3 Identification of PRGs with differential expression

TCGA‐LIHC cohort was investigated for genes showing distinct expressions between tumor and non‐tumor tissues. The “DESeq2” R package was used, with a threshold of |log2Foldchange (log2FC) | > 0.1 and false discovery rate (FDR) <0.05, and instances with insufficient gene expression data were excluded.

### 2.4 Detection of the PRGs‐based molecular categorization of TCGA‐LIHC patients

To classify the TCGA‐LIHC patients into distinct molecular subtypes based on differentially expressed PRGs (DEPRGs), we employed an unsupervised consensus clustering approach. This approach utilized the *k*‐means algorithm, implemented through the ConsensusClusterPlus package.^24^ The process of consensus clustering was meticulously designed to ensure robustness and reproducibility of the resulting subtypes.

Specifically, the clustering was conducted over 1000 bootstrap iterations, with each iteration randomly sampling 80% of the patient data. This bootstrapping approach enhances the stability and reliability of the clustering outcomes. To determine the most appropriate number of clusters (i.e., molecular subtypes), we relied on a comprehensive evaluation of several metrics:

1. The relative change in area under the CDF Curves: This metric evaluates the rate of change in cluster stability as the number of clusters increases. A smaller rate of change suggests a more stable cluster configuration.
2. The proportion of ambiguous clustering (PAC) Algorithm: PAC quantifies the degree of uncertainty or ambiguity in assigning samples to clusters. A lower PAC value indicates clearer distinction between clusters.
3. Consensus heatmap analysis^25^: By visualizing the consensus matrix as a heatmap, we assessed the clustering's consistency. The heatmap provides a qualitative measure of the clustering quality, where distinct blocks of high consensus indicate well‐defined clusters.

By integrating these methods, we determined the optimal number of molecular subtypes rooted in pyroptosis‐related gene expression patterns among TCGA‐LIHC patients. This comprehensive and systematic approach ensures that the molecular categorization is robust, reproducible, and reflective of underlying biological differences associated with pyroptosis in liver cancer.

### 2.5 Gene set variation analysis (GSVA)

To analyze significantly enriched molecular pathways of pyroptosis subtypes, the GSVA package was used.^26^ Furthermore, differential analysis of enrichment scores for Kyoto Encyclopedia of Genes and Genomes (KEGG) pathways was conducted with the limma package,^27^ identifying KEGG pathways with |log2FC| > 0.2 and FDR <0.05 as the most differentially enriched pathways between pyroptosis subtypes.^28^

### 2.6 The immunogenomic features of TCGA‐LIHC patients

Based on the gene expression profiles of TCGA‐LIHC samples, we analyzed 29 immune‐associated gene sets which represented the comprehensive immune activity of tumors, encompassing the types, functionalities, and molecular pathways of tumor infiltrating immune cells.^29^ Single‐sample gene‐set enrichment analysis (ssGSEA) was used for quantification of immune gene sets' enrichment.^26^

### 2.7 Development and validation of the pyroptosis subtype‐based prognostic model

To distinguish the differentially expressed genes (DEGs) between the pyroptosis subtypes, we examined the TCGA‐LIHC cohort using the “DESeq2” R package, ensuring FDR <0.05 and |log2FC| > 1. We evaluated the predictive significance of DEGs in the cohort using random forest Cox regression analyses. Univariate Cox regression analyses with a *p*‐value cutoff of 0.2 were then employed to assess the prognostic significance. We employed the LASSO‐Cox regression model with the glmnet package to screen the candidate genes and construct a predictive model. This process identified the key pyroptosis subtype‐related genes (PSRGs) for predicting prognosis in patients with HCC. The risk score, denoted as “pyroptosis subtype‐related score (PSRS),” was then computed in the TCGA‐LIHC cohort. The PSRS score was calculated using the following formula:

$$\text{PSRS score}=\sum_{k-1}^{n}X_{k}\times Y_{k}\;\left(X:\text{coefficients},Y:\text{gene expression level}\right)$$

Patients with HCC were classified into the high‐PSRS and low‐PSRS groups using the median PSRS, and the difference in overall survival (OS) between the two groups was analyzed. To validate the findings, the patients with HCC in LIRI‐JP and CHCC‐HBV datasets was also analyzed, where the formula was utilized to compute the PSRS score in these cohorts. Patients with HCC were then classified into high‐PSRS and low‐PSRS categories based on the median risk score, and their OS rates were compared. Receiver operating characteristic (ROC) curves were generated using the “timeROC,” “survminer” and “survival” R packages. To determine whether the PSRS serves as an independent prognostic factor, both univariate and multivariate Cox regression analyses were performed, taking into account covariates such as age, gender, grade, stage, and alpha‐fetoprotein (AFP).

### 2.8 Construction of nomogram and calibration curves

The nomogram was constructed to predict individual survival probability using the “RMS” package. We also generated calibration curves to predict the 1‐, 3‐, and 5‐year survival rates of patients with HCC.

### 2.9 Drug sensitivity analysis

Using the oncoPredict package of R software, the sensitivity score of each small molecular compound was computed for every patient in the high‐PSRS and low‐PSRS groups.

### 2.10 Statistical analyses

The study employed the independent Student's *t*‐test and Chi‐squared test for continuous and categorical data, respectively, to conduct pairwise comparisons across groups. The Mann–Whitney *U*‐test was used to evaluate gene expression, immune cell infiltration, and immune pathway activation between nontumor and tumor tissues, while the Kruskal–Wallis test was used for comparisons across multiple groups. R 4.1.3 was used for all statistical analyses, and a two‐tailed *p*‐value of less than 0.05 was considered statistically significant unless stated otherwise. Odds ratios (ORs), hazard ratios (HRs), and 95% confidence intervals (CIs) were reported when necessary.

## 3 RESULTS

### 3.1 Identification of differentially expressed PRGs in TCGA‐LIHC patients

**FIGURE 1:**
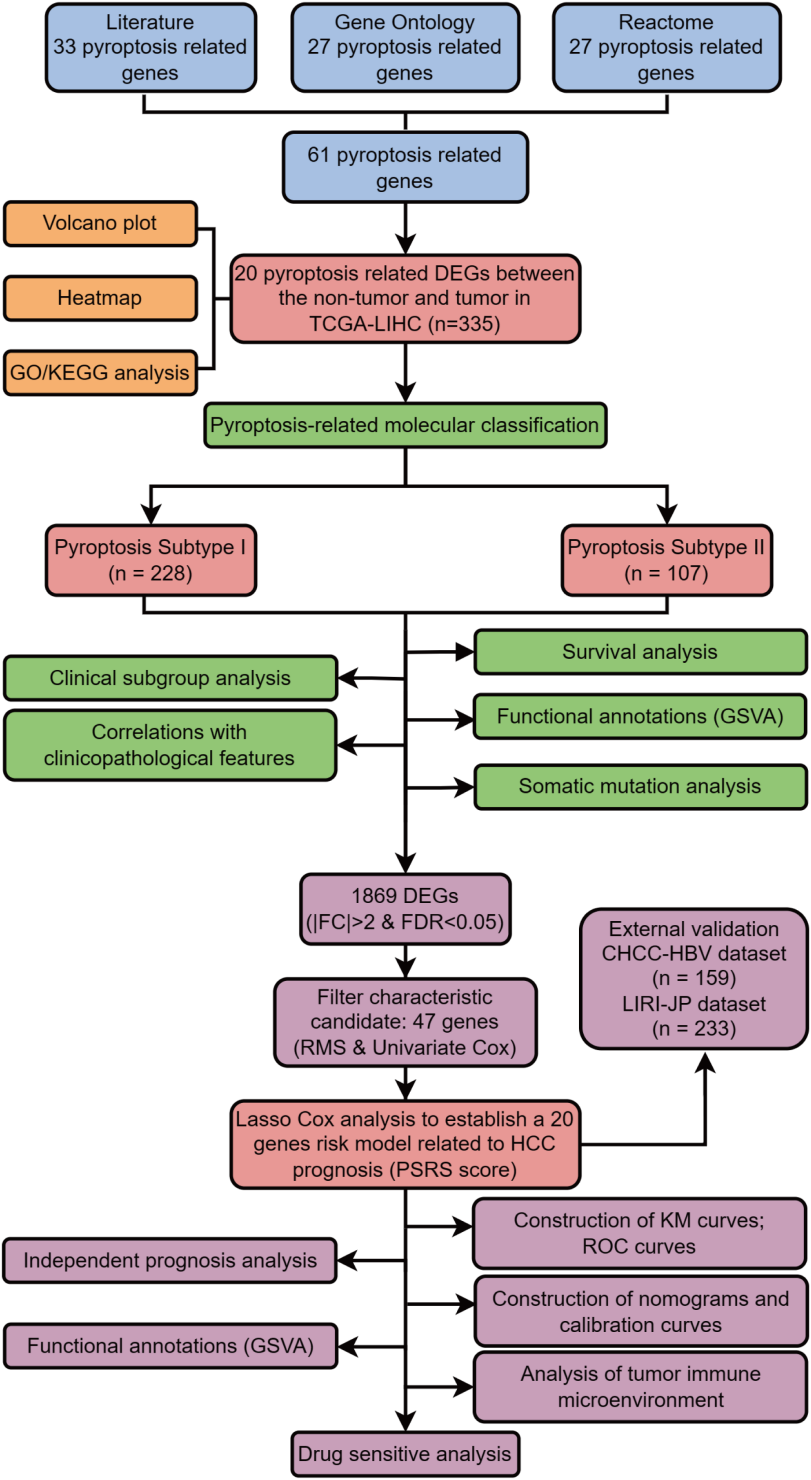
The flowchart of our research process.

**FIGURE 2:**
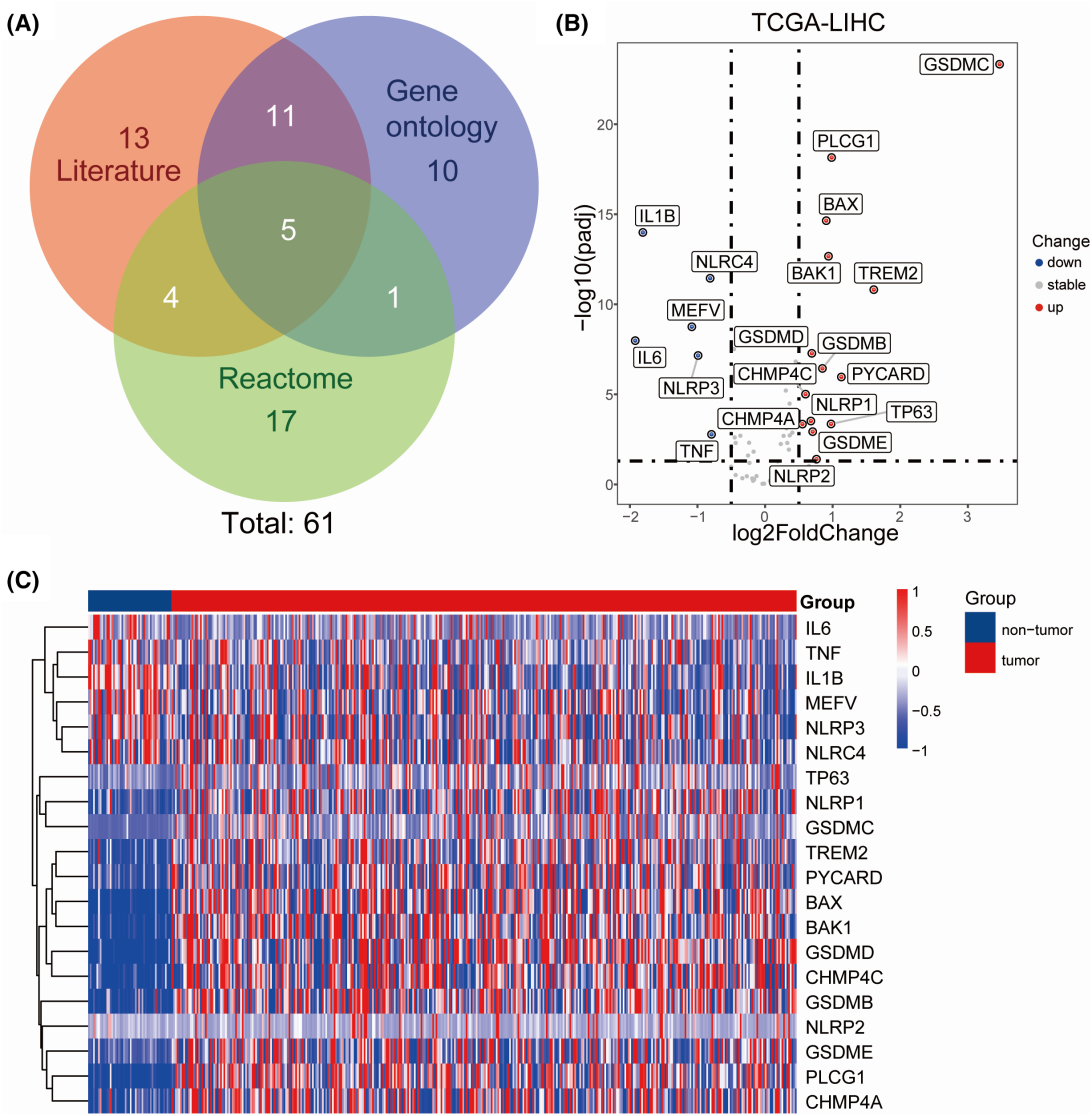
Differentially expressed pyroptosis‐related genes between HCC tissues and normal tissues. (A) pyroptosis‐related genes detected from three parts. (B) Volcano plot indicates pyroptosis‐related genes, with red dots indicating high expression and blue dots indicating low expression. (C) Heatmap of differentially expressed pyroptosis‐related genes, with red indicating high expression, blue indicating low expression.

The study's workflow is shown in Figure 1. First, 33 PRGs were extracted from previous researches.^16^, ^20^, ^21^, ^22^, ^23^ Besides, 27 and 27 genes were identified respectively in the GOBP_PYROPTOSIS and REACTOME_PYROPTOSIS gene sets. After the removal of duplicated genes from the three gene sets, 61 genes were defined as PRGs (Figure 2A). The expression of these 61 PRGs were evaluated in non‐tumor and tumor samples in the TCGA‐LIHC dataset. By the criteria of FDR <0.05 and |log2FC| > 0.5, 20 DEPRGs were identified. Among them, 14 genes were upregulated, while 6 genes were downregulated in TCGA‐LIHC samples, as demonstrated by volcano plots and heatmaps (Figure 2B,C).

### 3.2 Functional enrichment analysis

To better understand the functions of the DEPRGs, GO and KEGG analyses were conducted using the clusterProfiler package.^30^, ^31^, ^32^ The GO enrichment analysis indicated that the DEPRGs were primarily associated with the regulatory processes that control interleukin‐1 beta production, inflammasome complex, and phosphatidylserine binding, etc (Figure S1A). Furthermore, the KEGG analysis demonstrated that these DEPRGs participated in a variety of biological processes, including NOD‐like receptor signaling pathway, necroptosis, apoptosis, etc (Figure S1B). These results suggest that DEPRGs are involved in numerous biological pathways beyond pyroptosis.

### 3.3 Identification of two pyroptosis subtypes characterized by distinct survival outcomes, functional annotations, and clinical features

**FIGURE 3:**
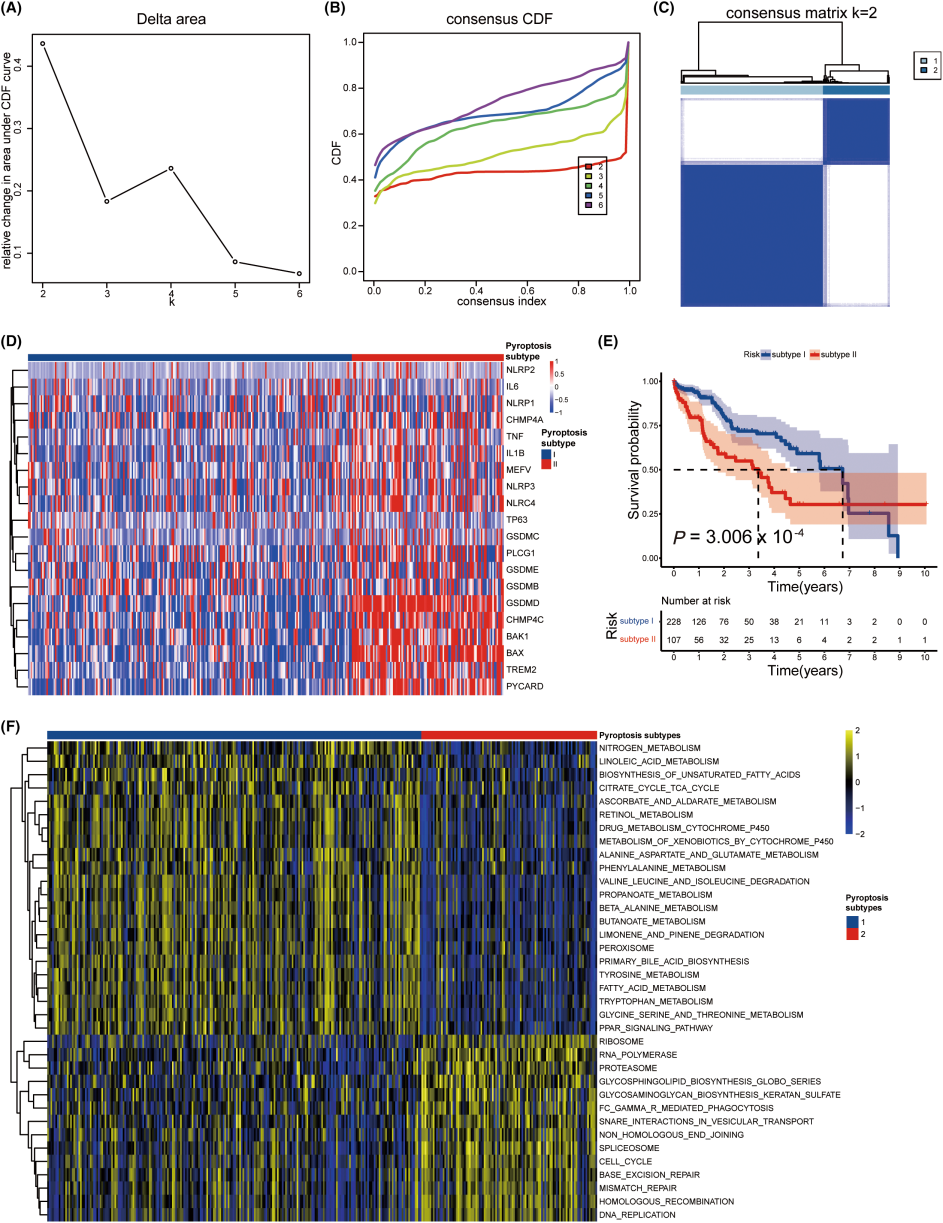
Identification of two pyroptosis subtypes with distinct survival outcomes and functional annotations. (A) Consensus clustering matrix for *k* = 2, which was the optimal cluster number. (B) CDF curves of the consensus score from *k* = 2 to 6. (C) The relative change in the area under the CDF curve from *k* = 2 to 6. (D) The heatmap of the expression patterns of 20 DEGs, with red indicating high expressions and blue indicating low expressions. The upper columns were the pyroptosis subtype of patients with HCC. (E) Kaplan–Meier survival analysis exhibited significantly better OS in patients with Pyroptosis Subtype I. (F) Heatmap illustrated the enrichment scores of 36 differentially enriched molecular pathways evaluated by GSVA analysis between Pyroptosis Subtypes I and II. Yellow represented high enrichment scores, and blue represented low enrichment scores.

The expression patterns of 20 DEPRGs in TCGA‐LIHC patients were analyzed using unsupervised consensus clustering to explore a new molecular characterization. Based on the relative change in the area under the CDF curve, the PAC algorithm, and the consensus heatmap, the optimal number of clusters was determined to be two (*k* value = 2) (Figure 3A–C). All patients with HCC were classified into two subgroups: Pyroptosis Subtype (PS) I (228 patients, 68.1%) and PS II (107 patients, 31.9%) (Figure 3D). The results of K–M survival analysis showed that PS I patients had significantly enhanced OS compared to those in the PS II group (HR = 0.473, log‐rank *p* = 3.006 × 10^−4^) (Figure 3E). The median OS time of patients in the PS I group was longer than that of the PS II group (6.73 vs. 3.37 years). Univariate and multivariate Cox regression analyses were conducted to assess the prognostic significance of PS and various clinicopathological variables.^33^ The results of these analyses showed that the PS was significantly correlated with OS in the TCGA‐LIHC cohort (Table 1), demonstrating that the PS is an independent prognostic factor.

**TABLE 1:** Univariate and multivariate cox proportional hazards analysis of clinicopathological variables and PS for overall survival in the TCGA‐LIHC cohort.

	TCGA‐LIHC(*n* = 260)			
	Univariate analysis		Multivariate analysis	
	HR (95% CI)	*p*‐value	HR (95% CI)	*p*‐value
Age (older vs. young)	1.66 (1.00–2.77)	**0.052**	1.67 (0.98–2.84)	0.059
Gender (female vs. male)	1.49 (0.89–2.47)	**0.127**	1.30 (0.77–2.22)	0.329
Grade (G3/G4 vs. G1/G2)	1.51 (0.91–2.5)	**0.113**	1.28 (0.76–2.17)	0.357
Stage (stage III/IV vs. stage I/II)	1.44 (0.82–2.55)	0.205	–	–
AFP (≥400 vs. <400)	0.89 (0.49–1.61)	0.691	–	–
PS (II vs. I)	2.13 (1.29–3.50)	**0.003**	2.11 (1.26–3.54)	**0.005**

*Note*: All statistical tests were two sided. Bold type means significant.

Abbreviations: AFP, alpha‐fetoprotein; CI, confidence interval; HR, hazard ratio; PS, pyroptosis subtype.

Furthermore, GSVA was carried out to gain insight into the molecular pathways and underlying mechanisms associated with the pyroptosis subtypes of TCGA‐LIHC patients. Thirty‐six molecular pathways that were differentially enriched were identified, comprising of 22 pathways positively correlated with PS I while 14 pathways positively associated with PS II (Figure 3F). PS I tumors were primarily correlated with the biosynthesis of unsaturated fatty acids, peroxisome function, fatty acid metabolism, and the PPAR signaling pathway, while PS II tumors were mostly associated with the cell cycle, DNA replication, mismatch repair, and spliceosome function.

Subsequently, a comparison of the demographic and clinicopathological characteristics of TCGA‐LIHC patients between the PS I and II groups was also conducted (Table 2). The distribution of OS status, tumor grade, and AFP level were all markedly different between the two subtypes. However, there was no significant difference in the age, gender, tumor stage, Child‐Pugh classification grade, and fibrosis Ishak score distribution of patients with HCC between the two subtypes.

**TABLE 2:** Demographics and clinicopathological features of HCC patients in the TCGA‐LIHC cohort.

Variables			*p*‐value
	PS I (*n* = 228)	PS II (*n* = 107)	
Age (years)	60.3 ± 13.6	59.3 ± 12.1	0.244
Gender	‐	‐	0.373
Male	147 (64.5%)	75 (70.1%)	
Female	81 (35.5%)	32 (29.9%)	
OS status	‐	‐	1.162 × 10^−3^
Alive	166 (72.8%)	58 (54.2%)	
Dead	62 (27.2%)	49 (45.8%)	
Child‐Pugh classification grade	‐	‐	0.674
A	148 (90.2%)	66 (93.0%)	
B	16 (9.8%)	5 (7.0%)	
C	1 (−)	0 (−)	
NA	63 (−)	36 (−)	
Tumor stage		‐	0.745
Stage I	115 (55.3%)	51 (49.5%)	
Stage II	50 (24.0%)	27 (26.2%)	
Stage III	40 (19.2%)	24 (23.3%)	
Stage IV	3 (1.4%)	1 (1.0%)	
NA	20 (−)	4 (−)	
Grade	‐	‐	3.384 × 10^−3^
G1	37 (16.4%)	10 (9.5%)	
G2	116 (51.6%)	43 (41.0%)	
G3	68 (30.2%)	44 (41.9%)	
G4	4 (1.8%)	8 (7.6%)	
NA	3 (−)	2 (−)	
Fibrosis Ishak Score	‐	‐	0.101
0—No fibrosis	56 (37.3%)	16 (29.1%)	
1,2—Portal fibrosis	20 (13.3%)	11 (20.0%)	
3,4—Fibrous speta	17 (11.3%)	9 (16.4%)	
5—Nodular formation and incomplete cirrhosis	4 (2.7%)	5 (9.1%)	
6—Established cirrhosis	53 (35.3%)	14 (25.5%)	
NA	78 (−)	52 (−)	
AFP	‐	‐	9.113 × 10^−5^
<400	157 (84.0%)	53 (61.6%)	
≥400	30 (16.0%)	33 (38.4%)	
NA	41 (−)	21 (−)	

Abbreviations: AFP, alpha‐fetoprotein; NA, not available; OS, overall survival; PS, pyroptosis subtype.

### 3.4 The pyroptosis subtypes possessed distinct somatic mutation pattern

**FIGURE 4:**
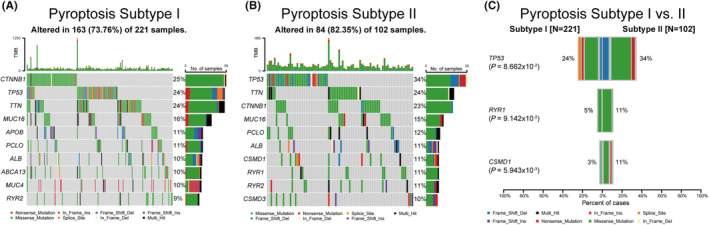
Comparisons of somatic variations between Pyroptosis Subtypes I and II. Waterfall plots showed the top 10 mutated in Pyroptosis Subtypes I (A) and II (B). (C) Most differentially mutated genes between HCC pyroptosis subtypes in TCGA‐LIHC cohort.

Previous studies have investigated the role of genomic alterations in influencing tumor immunity and immune infiltration profiles.^34^, ^35^ Hence, somatic mutational analysis was conducted to explore the distinct genomic variations between the two pyroptosis subtypes and revealed specific highly mutated genes in each pyroptosis subtype (Figure 4A,B). *TP53* was the most frequently mutated gene in PS II (34%), whereas *CTNNB1* was the most commonly mutated gene in PS I (25%). The somatic mutation analysis also identified three differentially mutated genes out of 13 genes after merging the top 10 mutated genes from each subtype. *TP53* mutations were more frequent in the PS II cohort compared to the PS I cohort (34% vs. 24%; *p* = 8.662 × 10^−2^), which is similar to the findings of *RYR1* and *CSMD1* mutations. The frequencies of *RYR1* (11% vs. 5%; *p* = 9.142 × 10^−2^) and *CSMD1* (11% vs. 3%; *p* = 5.943 × 10^−3^) mutations in the PS II group were significantly higher than those in the PS I group (Figure 4C).

### 3.5 Construction of a prognostic model using PRGs in the training set

**FIGURE 5:**
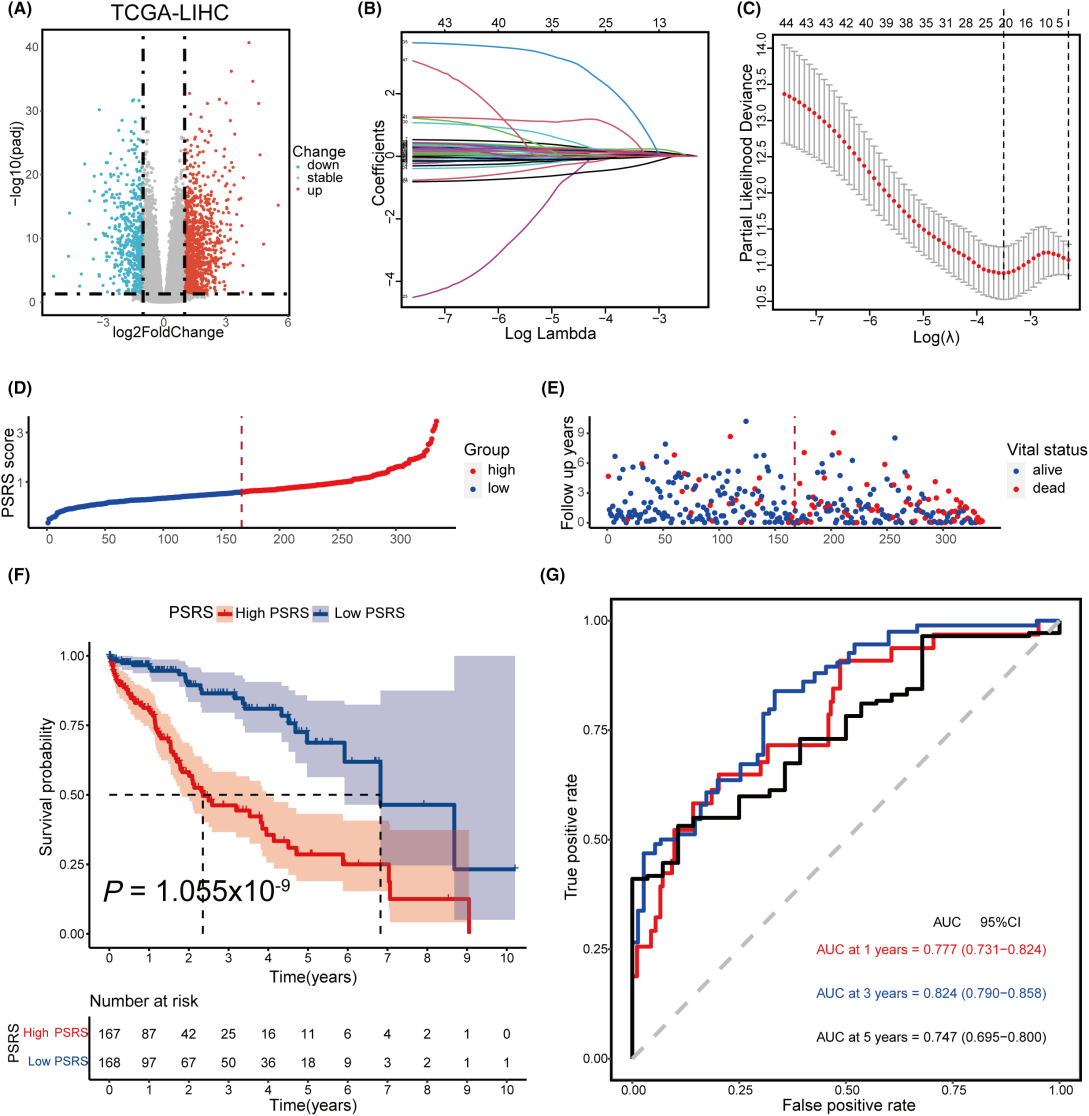
Construction of a risk prognostic model based on pyroptosis subtype‐related genes in the TCGA cohort. (A) Volcano plot indicates pyroptosis‐related genes, with red dots indicating high expression and blue dots indicating low expression in Pyroptosis Subtype II. (B) LASSO regression of the 20 OS‐related genes. (C) Cross‐validation for tuning the parameter selection in the LASSO regression. (D) The patients were equally divided into two groups according to the threshold of the median PSRS. Blue represents the low‐PSRS group. Red represents the high‐PSRS group. (E) Survival status of patients with HCC in high and low PSRS groups. Blue represents survival. Red represents death. (F) Kaplan–Meier curves showing the OS of patients in the high‐PSRS and low‐PSRS groups. (G) The predictive efficiency of the PSRS was verified by the ROC curve.

As shown in Figure 5A, we filtered 1869 DEGs in the two pyroptosis subtypes, using the “DESeq2” R package, with a threshold of |log2FC| > 1 and FDR <0.05. We preserved a set of 90 characteristic genes after screening them further through the random forest algorithm. We filtered out 47 of the 90 genes with a *P*‐value of less than 0.2 through univariate Cox regression analysis. Subsequently, we constructed a pyroptosis‐related prognostic model involving 20 genes through LASSO regression (Figure 5B,C).

This method allowed us to derive coefficients for each of the PRGs, thus constructing the PSRS. The formula for calculating the PSRS for each patient is as follows: PSRS = (−0.026 × *IGFALS* expression level (exp.)) + (0.096 × *HAVCR1* exp.) + (0.151 × *STAC* exp.) + (0.167 × *SMOX* exp.) + (0.114 × *GAL* exp.) + (0.002 × *GAGE1* exp.) + (0.158 × *TFDP3* exp.) + (0.234 × *RNF186* exp.) + (0.079 × *P2RY6* exp.) + (0.122 × *GCG* exp.) + (0.555 × *DAW1* exp.) + (−0.332 × *SIRPG* exp.) + (0.072 × *PRAC2* exp.) + (0.069 × *NELL1* exp.) + (0.038 × *MSC* exp.) + (0.001 × *MACIR* exp.) + (1.437 × *LOXHD1* exp.) + (0.092 × *EPO* exp.) + (0.122 × *PYDC1* exp.) + (−0.047 × *PPARGC1A* exp.). This formula provides a PSRS for each patient in the TCGA‐LIHC dataset. We subsequently categorized the entire cohort into two groups based on the median value of the PSRS. This resulted in a high‐PSRS group (*n* = 167) and a low‐PSRS group (*n* = 168), enabling us to compare prognostic outcomes between these two cohorts more effectively (Figure 5D). The high‐PSRS group showed higher mortality incidence and shorter OS time compared to the low‐PSRS group (Figure 5E,F). The ROC analysis indicated that the accuracy of OS prognostics was 0.777 at 1 year (95% CI 0.731–0.824), 0.824 at 3 years (95% CI 0.790–0.858), and 0.747 at 5 years (95% CI 0.695–0.800) (Figure 5G). These findings suggest that our model shows high accuracy in predicting prognosis in the training set.

### 3.6 Validation of the prognostic model in the test sets

To assess the accuracy of the prognostic model we developed, we applied the identical formula to calculate PSRS in 233 patients with HCC from the LIRI‐JP cohort. Based on the median PSRS, 117 patients were categorized into the low‐PSRS group, and 116 patients into the high‐PSRS group (Figure S2A). The high‐PSRS group demonstrated higher mortality incidence and shorter OS time than the low‐PSRS group (Figure S2B,C), which was consistent with the result of the training set. The ROC analysis revealed that the prognostic accuracy of OS was 0.727 at 1 year (95% CI 0.665–0.788), 0.663 at 2 years (95% CI 0.607–0.719), and 0.716 at 3 years (95% CI 0.662–0.771) (Figure S2D). Similarly, we computed PSRS for 159 patients with HCC from the CHCC‐HBV cohort using the same formula as in the training set. Based on the median PSRS, 79 patients were categorized into the low‐PSRS group, while the remaining 80 patients were placed in the high‐PSRS group (Figure S3A). The patients in the high‐PSRS group had higher mortality incidence and shorter OS time than those in the high‐PSRS group (Figure S3B,C), which was consistent with the observations made in the training set. The ROC analysis demonstrated that the prognostic accuracy of OS was 0.716 at 1 year (95% CI 0.654–0.778), 0.680 at 2 years (95% CI 0.633–0.726), and 0.606 at 3 years (95% CI 0.554–0.658) (Figure S3D). These results further confirm the accuracy of our established prognostic model.

### 3.7 Independent prognosis analysis of PSRS and clinical characteristics

We conducted a comparative analysis of the PSRS among various clinicopathological characteristic groups in TCGA‐LIHC patients. Figure S4A shows a positive correlation between the PSRS and grade (*p* = 1.256 × 10^−4^) and stage (*p* = 8.174 × 10^−5^), while no significant difference was observed in the other four groups (age, gender, Child‐Pugh grade, and AFP). To evaluate the potential of PSRS and clinical features as independent prognostic factors, we performed univariate and multivariate analyses. The results of the univariate independent prognosis analysis showed a significant correlation between age, gender, grade, and PSRS with the OS (Figure S4B). Moreover, the multivariate independent prognosis analysis revealed that the PSRS could independently predict the prognosis of TCGA‐LIHC patients (*p* < 0.001) (Figure S4C).

### 3.8 Construction of nomogram and calibration curves

**FIGURE 6:**
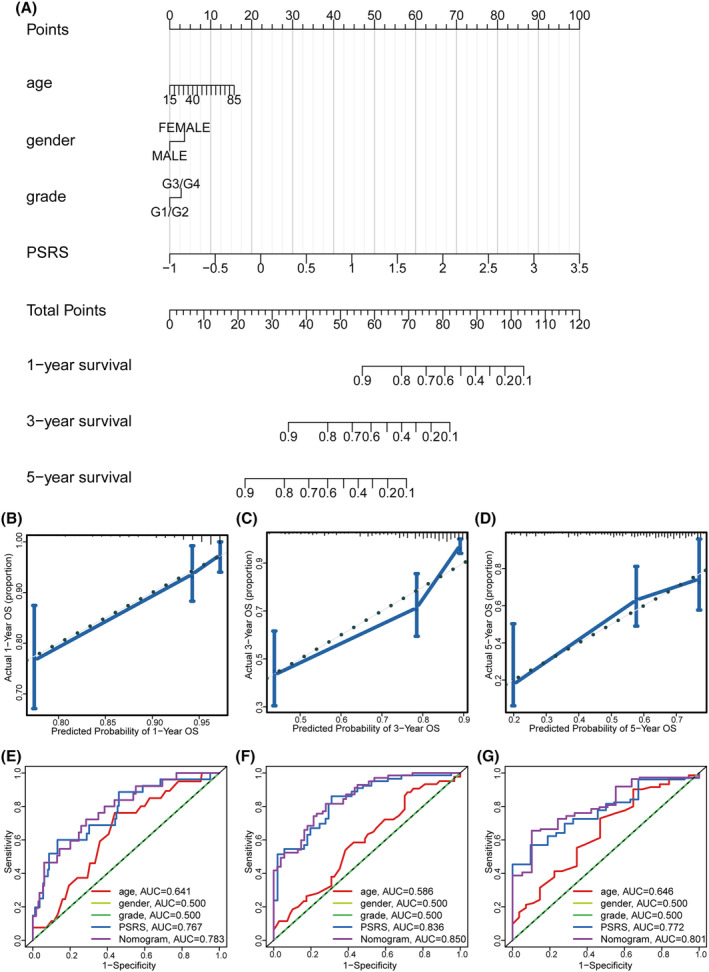
Nomogram to predict survival probability of TCGA‐LIHC patients. (A) Nomogram combining PSRS with pathologic features. Calibration plots for predicting 1‐ (B), 3‐ (C), 5‐year (D) OS of patients. 1‐ (E), 3‐ (F), 5‐year (G) ROC curves for prediction of survival by nomogram model, the PSRS, and other variables (age, gender, grade).

To offer physicians with a better quantitative method to predict the prognosis of the patients with HCC, we established a nomogram model that combined demographic and clinical variables including age, gender, tumor grade, tumor stage, and PSRS based on TCGA‐LIHC patients. According to Figure 6A, the PSRS emerged as a significant factor among various demographic and clinical variables in the prognostic model. Calibration curves demonstrated that the prognostic model had a significant correlation with the actual survival data of TCGA‐LIHC patients (Figure 6B–D). Our nomogram model demonstrated significantly superior performance in comparison to traditional prognostic scoring systems, with consistently high areas under the ROC curve (AUC) values for 1, 3, and 5 years (Figure 6E–G). Thus, our study underscores that the nomogram model using PSRS provides an accurate prediction of the OS of patients with HCC.

### 3.9 Functional annotations in the high‐PSRS and low‐PSRS groups

GSVA enrichment analysis was conducted separately in the high‐ and low‐PSRS groups in both the training and validation sets. Our findings revealed that in the training set, the low‐PSRS group had enriched pathways such as beta‐alanine metabolism, valine, leucine and isoleucine degradation, propanoate metabolism, fatty acid metabolism, limonene and pinene degradation, glycine, serine, and threonine metabolism, and primary bile acid biosynthesis (Figure S5A). Likewise, similar enriched pathways were also observed in the low‐PSRS group in the validation set (LIRI‐JP and CHCC‐HBV). Furthermore, the high‐PSRS group showed enriched pathways in DNA replication, cell cycle, and mismatch repair (Figure S5B,C).

### 3.10 Analysis of the tumor microenvironment in the high‐PSRS and low‐PSRS groups

We conducted ssGSEA on 29 immune signatures related to PSRS in TCGA‐LIHC patients to investigate the underlying mechanisms. Among these, 14 signatures exhibited differential enrichment. Particularly, six anti‐tumor signatures, including effector cells, effector cell traffic, NK cells, T cells, Th1 signature, and antitumor cytokines, were significantly enriched in the low‐PSRS group. Conversely, eight pro‐tumor signatures, including granulocyte traffic, myeloid cells traffic, protumor cytokines, cancer‐associated fibroblasts, matrix, matrix remodeling, angiogenesis, and tumor proliferation rate, were highly enriched in the high‐PSRS group (Figure S6). The findings suggest that the low‐PSRS group mainly displayed a higher abundance in anti‐tumor signatures, while the high‐PSRS group exhibited increased enrichment in pro‐tumor signatures. These results may offer insights into the differential prognosis associated with PSRS in TCGA‐LIHC patients.

### 3.11 Evaluating the therapeutic response in the high‐PSRS and low‐PSRS groups

Using the oncoPredict algorithm on Genomics of Drug Sensitivity in Cancer (GDSC) database, we estimated chemotherapeutic response through half‐maximal inhibitory concentration (IC50) data for TCGA‐LIHC patients with high and low PSRS. Our findings identified 65 small molecular compounds with significantly different responses between the two groups (Table S2). Among these, the top four with lowest *p* values between high‐PSRS and low‐PSRS were Dasatinib_1079, IAP_5620_1428, SB505124_1194, and TAF1_5496_1732 (Figure S7A–D). Our findings suggest these small molecular compounds as potential agents for treating patients with HCC, with IAP_5620_1428, SB505124_1194, and TAF1_5496_1732 being more effective in low‐PSRS tumors, and Dasatinib_1079 better suitable for high‐PSRS tumors. Further analysis is necessary to determine the efficacy of these compounds as HCC treatment agents. In a word, our results provide potential molecular chemotherapy compounds for patients with HCC.

## 4 DISCUSSION

Our study aimed to explore pyroptosis subtypes based on PRGs and establish a prognostic model to predict the prognosis of patients with HCC. In recent years, the search for prognostic markers and prognostic models of HCC has garnered significant research attention.^36^, ^37^, ^38^, ^39^, ^40^ These models present prognostic implications that would largely benefit patients with HCC for the prediction of the prognosis. Consistent with previous studies, the prognostic model we have formulated has shown commendable efficacy in predicting the prognosis of patients with HCC.

In our study, we analyzed the expression of 61 genes, reported in the literature and databases as PRGs, in HCC and non‐tumor samples of the TCGA‐LIHC dataset. The results indicated that 20 of these PRGs exhibited differential expression. Then, we applied consensus clustering, a widely used unsupervised clustering method,^41^ to stratify TCGA‐LIHC patients based on the expression of 20 DEPRGs. Furthermore, we compared the clinicopathological parameters to establish the relationship between pyroptosis subtypes and clinical characteristics. Our results suggest that PS II subtype patients exhibited a significantly higher level of PRGs expression, poorer prognosis.

Our study of the signaling pathways related to the two subtypes of pyroptosis in HCC reveals potential mechanisms that contribute to their differing prognoses. Specifically, in the PS I subtype, the activation of pathways such as the PPAR signaling pathway highlights its significant involvement in the regulation of liver metabolism. Dysregulation of PPARα and PPARγ has been strongly associated with the development of HCC. PPARγ has been identified as a tumor‐suppressor gene in hepatocarcinogenesis, exerting its inhibitory effects on tumor cell growth through mechanisms such as suppression of cell proliferation, induction of G2/M arrest, and promotion of apoptosis.^42^ Activation of the peroxisome pathway has been associated with a more favorable prognosis for patients with HCC, with studies indicating that individuals with low peroxisomal function, particularly those with HCV‐related HCC, are at increased risk of adverse clinical outcomes.^43^ On the other hand, the PS II subtype exhibits activation of pathways related to cell cycle regulation, DNA replication, mismatch repair, and spliceosome function. The dysregulation of cell cycle regulation is frequently associated with the uncontrolled growth of HCC cells. Studies have shown a correlation between the autophagic degradation machinery and the cell‐cycle regulator cyclin D1 in the pathogenesis of HCC tumors.^44^ Additionally, the upregulation of MCM7, a crucial component in DNA replication initiation, is predominantly detected in HCC tumors and is strongly associated with unfavorable prognostic outcomes in patients with HCC.^45^ These results are consistent with our own findings, indicating that distinct activation and inhibition of pathways may contribute to the prognostic differences between the two pyroptosis subtypes, warranting further exploration of the underlying mechanisms of gene expression within these pathways.

To facilitate the distinction between the two pyroptosis subtypes in clinical practice, we developed a novel prognostic model for patients with HCC using the 20 most important PSRGs. Furthermore, the model was further validated in two external cohorts. Additionally, we performed comprehensive analyses on the model scores (PSRS) and elucidated drug sensitivity analyses between the high‐PSRS and low‐PSRS groups. These results suggest that our analyses on the molecular subtypes are thorough, and the validated prognostic model is reliable and practical for clinical use and future research on HCC. Notably, the high‐PSRS group exhibited a considerably worse prognosis compared to the low‐PSRS group.

Regarding the mechanism, the impact of pyroptosis on the prognosis and progression of patients with HCC cannot be separated from tumor immunity, as our study suggests. Several immune‐related biological functions, such as viral protein interaction with cytokine and cytokine receptor, were significantly enriched. Hepatitis B and C viruses have been established as the two main risk factors for HCC,^46^ and they can affect HCC development in various ways, including inducing inflammation.^47^ Cytokines play a crucial role in promoting HCC carcinogenesis and progression, released in response to infection, inflammation, and carcinogen‐induced injury.^48^ Cytokine activity has also been identified as a key indicator of the severity and development of hepatitis B or C virus infections.^49^ According to the ssGSEA results in this study, the antitumor cytokine scores of the low‐PSRS group were found to be significantly higher than those of the high‐PSRS groups. Conversely, the protumor cytokine scores were observed to be higher in the high‐PSRS groups as compared to the low‐PSRS group. These findings suggest a potential correlation between the PSRS and the immune response, wherein the high‐PSRS group is associated with a predominant protumor cytokine milieu, while the low‐PSRS group exhibits a more pronounced antitumor cytokine expression profile. Further investigation is required to confirm and validate these observations in a larger cohort of subjects.

During the drug sensitivity analysis, a total of 65 small molecular compounds were identified to have significant differences in sensitivity between the two PSRS groups. Among these compounds, Dasatinib_1079, IAP_5620_1428, SB505124_1194, and TAF1_5496_1732 exhibited the most pronounced differences. Notably, each of these compounds has been linked to potential therapeutic effects for HCC. Dasatinib is a tyrosine kinase inhibitor used to treat certain types of leukemia. A previous study found that the axitinib, erlotinib, and dasatinib mixture inhibited tumor growth in both SW620 and HT29 CAM tumors.^50^ IAP‐5620 targets components such as TRAF2 and RIPK1, which have been associated with poor outcomes in HCC, suggesting its potential utility in this context.^51^ SB505124, impacting the TGF‐beta signaling pathway, presents a novel approach to targeting HCC, especially considering its association with ferroptosis and cancer metabolism.^52^ TAF1_5496_1732, by targeting TAF1, hints at the critical role of transcription regulation in HCC progression and offers a new angle for therapeutic intervention.^53^ These results highlight the potential of utilizing PSRS score‐based drug sensitivity analysis to discover novel therapeutic strategies for HCC. Although the identification of significant compounds with potential relevance to HCC treatment is promising, it is crucial to acknowledge the challenges associated with translating these findings into clinical applications. Additional research, encompassing experimental validation and clinical trials, is imperative to comprehensively evaluate the therapeutic efficacy and safety profiles of these compounds in the context of HCC.

In addition, our study has some limitations that should be considered in future researches. First, we only applied commonly used mRNA expression data for tumor classification in HCC subtyping. This leaves room for potential underestimation or overestimation of the subtypes, calling for more comprehensive data sources, such as gene mutations, to be considered in future investigations. Second, the lack of experimental validation of the model‐related genes leads to ambiguity about their functionality in HCC, which should be addressed in future studies. Last, due to the unavailability of a similar pyroptosis‐related prognostic model based on pyroptosis subtype, we could not undertake a comparative analysis to evaluate the superiority of our model. Therefore, future researches should aim to validate our model's effectiveness and explore its potential for further optimization.

## 5 CONCLUSION

Here, our investigation has demonstrated the close correlation between pyroptosis and HCC. Our study has established the PSRS as an independent risk factor for the prediction of HCC prognosis in the TCGA‐LIHC, LIRI‐JP, and CHCC‐HBV datasets based on the 20 pyroptosis subtype‐related genes. Our findings have also revealed the relationship between the PSRS and tumor immunity, as well as the identification of potential molecular compounds for HCC treatment. This study offers a promising direction for the identification of novel predictive markers for HCC prognosis and serves as a critical foundation for future investigations into the intricate relationship between pyroptosis and HCC immunity.

## AUTHOR CONTRIBUTIONS

**Haisheng Xie:** Conceptualization (lead); data curation (lead); formal analysis (equal); methodology (equal); software (lead); supervision (equal); writing – original draft (lead); writing – review and editing (equal). **Guanlin Huang:** Conceptualization (equal); data curation (equal); formal analysis (equal); methodology (equal); software (equal); validation (lead); writing – review and editing (equal). **Haoming Mai:** Formal analysis (equal); software (equal); validation (equal); writing – review and editing (equal). **Jiaxuan Chen:** Funding acquisition (equal); methodology (equal); validation (equal); writing – review and editing (equal). **Rong Na:** Validation (equal); writing – review and editing (equal). **De‐Ke Jiang:** Formal analysis (lead); funding acquisition (lead); methodology (lead); project administration (lead); resources (lead); supervision (lead); writing – review and editing (lead).

## FUNDING INFORMATION

This research was funded by the National Key Research and Development Program of China (No. 2022YFC2303600), the General Programs from the National Natural Science Foundation of China (No. 82272765, 81472618, 81670535, and 82203253), the Local Innovative and Research Teams Project of Guangdong Pearl River Talents Program (No. 2017BT01S131), the General Program from the Natural Science Foundation of Guangdong Province (No. 2022A1515220090, and 2019A1515011423), the Innovative Research Team Project of Guangxi Province (No. 2017GXNSFGA198002), the Dean Fund of Nanfang Hospital, Southern Medical University (No. 2018Z005), the Grant for Recruited Talents to Start Scientific Research from Nanfang Hospital, China, and the Outstanding Youths Development Scheme of Nanfang Hospital at Southern Medical University, China (No. 2017J001), and the Postdoctoral Science Foundation of China (No. 2022M711508).

## CONFLICT OF INTEREST STATEMENT

The authors declare no conflict of interest.

## ETHICS STATEMENT

This article does not contain any studies with human participants or animals performed by any of the authors.

## Supporting information


**Figure S1.** Functional enrichment analyses of gene ontology (GO) and Kyoto Encyclopedia of Genes and Genomes (KEGG). (A) Bar plot for GO enrichment (the longer bar means the more genes enriched). (B) Bubble graph for KEGG enrichment analysis of differentially expressed genes (the bigger bubble means the more genes enriched, and the increasing depth of red means the differences were more obvious).

**Figure S2.** Validation of the PSRS model in the LIRI‐JP cohort. (A) The patients were equally divided into two groups according to the threshold of the median PSRS score. Blue represents the low‐PSRS group. Red represents the high‐PSRS group. (B) Survival status of patients with LIHC in high and low PSRS groups. Blue represents survival. Red represents death. (C) Kaplan–Meier curves showing the overall survival of patients in the high‐PSRS and low‐PSRS groups. (D) The predictive efficiency of the PSRS score was verified by the ROC curve.

**Figure S3.** Validation of the PSRS model in the CHBB‐HBV cohort. (A) The patients were equally divided into two groups according to the threshold of the median PSRS score. Blue represents the low‐PSRS group. Red represents the high‐PSRS group. (B) Survival status of patients with LIHC in high and low PSRS groups. Blue represents survival. Red represents death. (C) Kaplan Meier curves showing the overall survival of patients in the high‐PSRS and low‐PSRS groups. (D) The predictive efficiency of the PSRS score was verified by the ROC curve.

**Figure S4.** The relationship with clinical characteristics and independent prognostic analysis of the PSRS in patients with HCC. (A) Violin plots of PSRS of the patients with HCC classified by age, gender, Child‐Pugh grade, stage, histological grade, and AFP levels. (B) Univariate independent prognosis Cox regression analysis of PSRS score and indicated clinical characteristics. (C) Multivariate independent prognosis Cox regression analysis of PSRS score and indicated clinical characteristics.

**Figure S5.** Functional annotations of low‐ and high‐PSRS group. Heatmap illustrated the enrichment scores of differentially enriched molecular pathways evaluated by GSVA analysis between low‐ and high‐PSRS group in the TGCA‐LIHC (A), LIRI‐JP (B), and CHBB‐HBV (C). Yellow represented high enrichment scores, and blue represented low enrichment scores.

**Figure S6.** Comparisons of the scores of 29 immune signatures in low‐ and high‐PSRS group.

**Figure S7.** The screened drugs for LIHC treatment. IC 50 value of Dasatinib_1079 (A), IAP_5620_1428 (B), SB505124_1194 (C), and TAF1_5496_1732 (D) in high‐and low‐PSRS patients with LIHC.

**Table S1.** Pyroptosis‐related genes.

**Table S2.** Small molecular compounds with significantly different responses between high‐PSRS and low‐PSRS groups.

## Data Availability

The study relies on publicly available data from the TCGA database, CHCC‐HBV dataset and ICGC database, which were used and analyzed. Further data are available from authors upon reasonable request.
