# Supplementary figures and images for: Identification of pyroptosis subtypes and prognosis model of hepatocellular carcinoma based on pyroptosis‐related genes
Source: Cancer Med. 2024 Aug 9;13(15):e70081. doi: 10.1002/cam4.70081 (PMC11316015; doi:10.1002/cam4.70081)

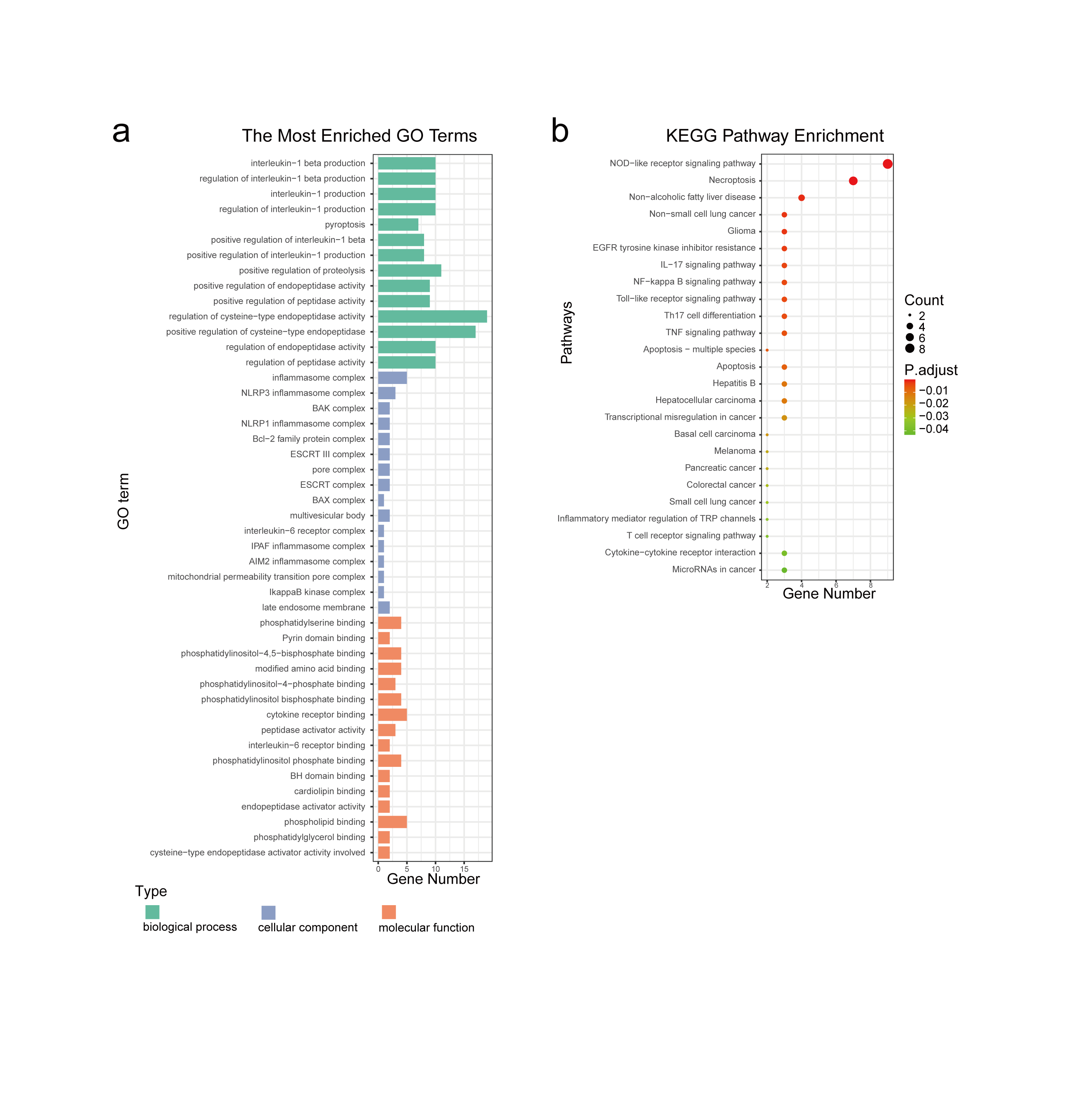

Supplement: Supplementary file 1 — Figure S1. Functional enrichment analyses of gene ontology (GO) and Kyoto Encyclopedia of Genes and Genomes (KEGG). (A) Bar plot for GO enrichment (the longer bar means the more genes enriched). (B) Bubble graph for KEGG enrichment analysis of differentially expressed genes (the bigger bubble means the more genes enriched, and the increasing depth of red means the differences were more obvious). [file CAM4-13-e70081-s003.tif]

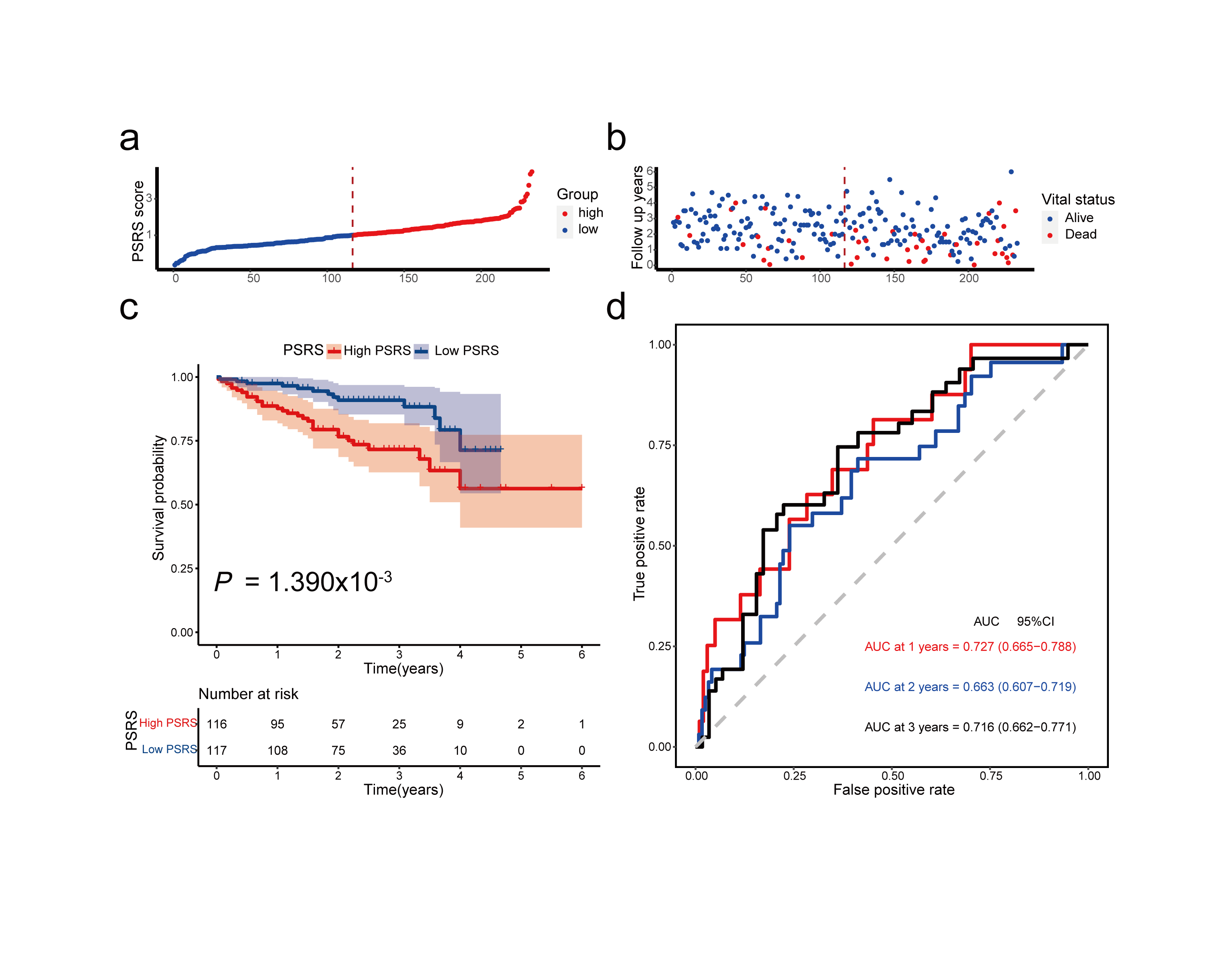

Supplement: Supplementary file 2 — Figure S2. Validation of the PSRS model in the LIRI‐JP cohort. (A) The patients were equally divided into two groups according to the threshold of the median PSRS score. Blue represents the low‐PSRS group. Red represents the high‐PSRS group. (B) Survival status of patients with LIHC in high and low PSRS groups. Blue represents survival. Red represents death. (C) Kaplan–Meier curves showing the overall survival of patients in the high‐PSRS and low‐PSRS groups. (D) The predictive efficiency of the PSRS score was verified by the ROC curve. [file CAM4-13-e70081-s009.tif]

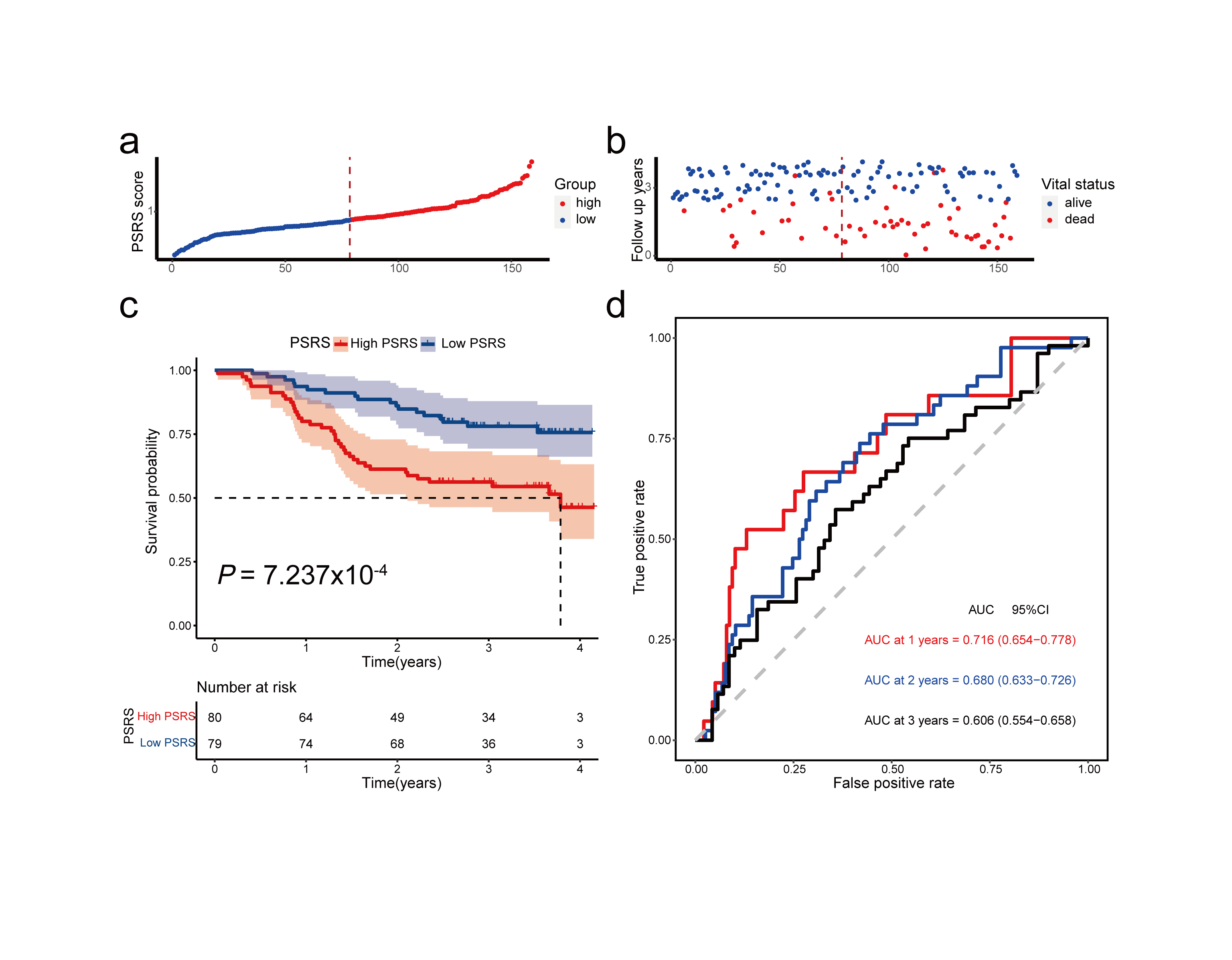

Supplement: Supplementary file 3 — Figure S3. Validation of the PSRS model in the CHBB‐HBV cohort. (A) The patients were equally divided into two groups according to the threshold of the median PSRS score. Blue represents the low‐PSRS group. Red represents the high‐PSRS group. (B) Survival status of patients with LIHC in high and low PSRS groups. Blue represents survival. Red represents death. (C) Kaplan Meier curves showing the overall survival of patients in the high‐PSRS and low‐PSRS groups. (D) The predictive efficiency of the PSRS score was verified by the ROC curve. [file CAM4-13-e70081-s007.tif]

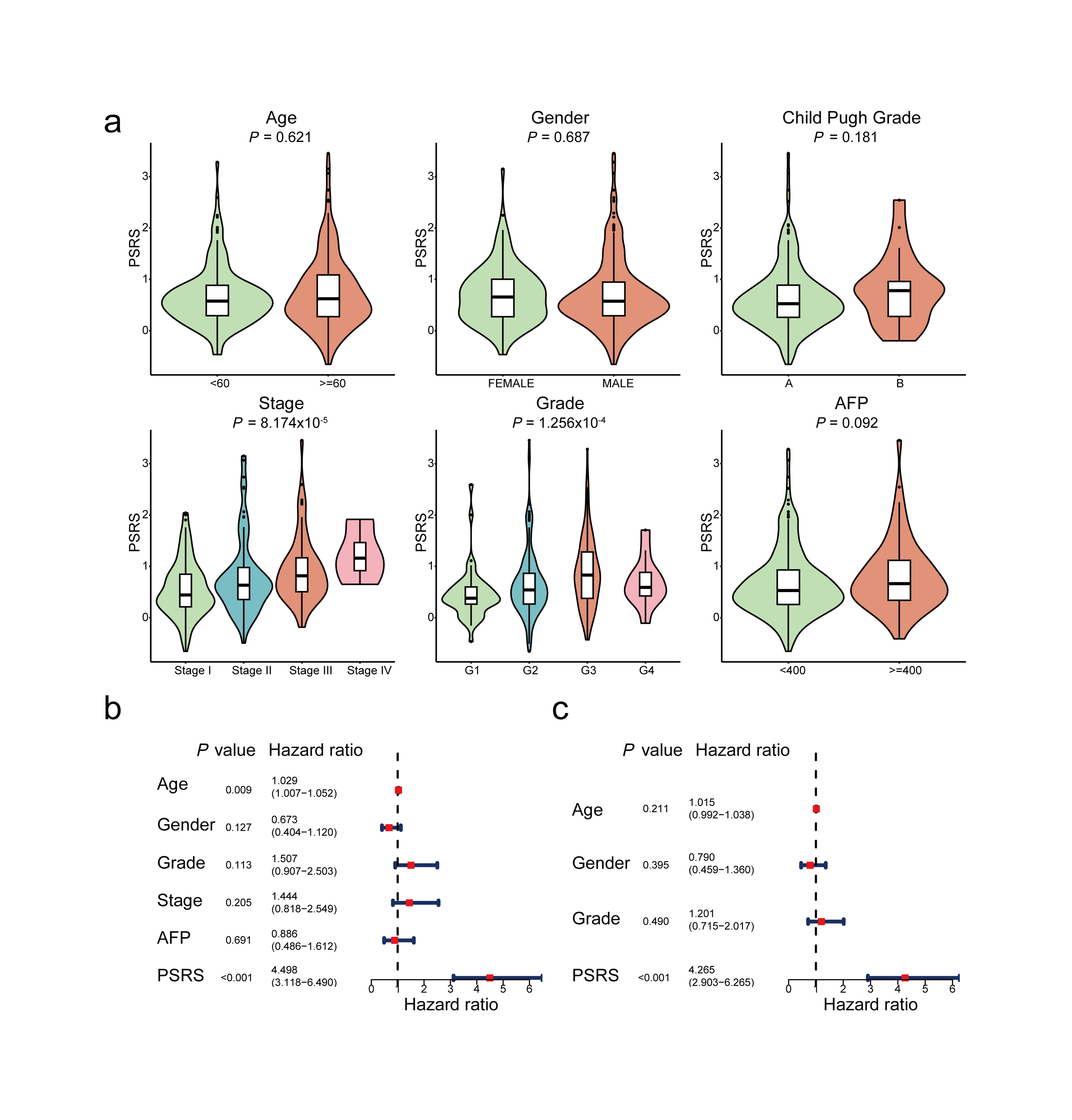

Supplement: Supplementary file 4 — Figure S4. The relationship with clinical characteristics and independent prognostic analysis of the PSRS in patients with HCC. (A) Violin plots of PSRS of the patients with HCC classified by age, gender, Child‐Pugh grade, stage, histological grade, and AFP levels. (B) Univariate independent prognosis Cox regression analysis of PSRS score and indicated clinical characteristics. (C) Multivariate independent prognosis Cox regression analysis of PSRS score and indicated clinical characteristics. [file CAM4-13-e70081-s005.tif]

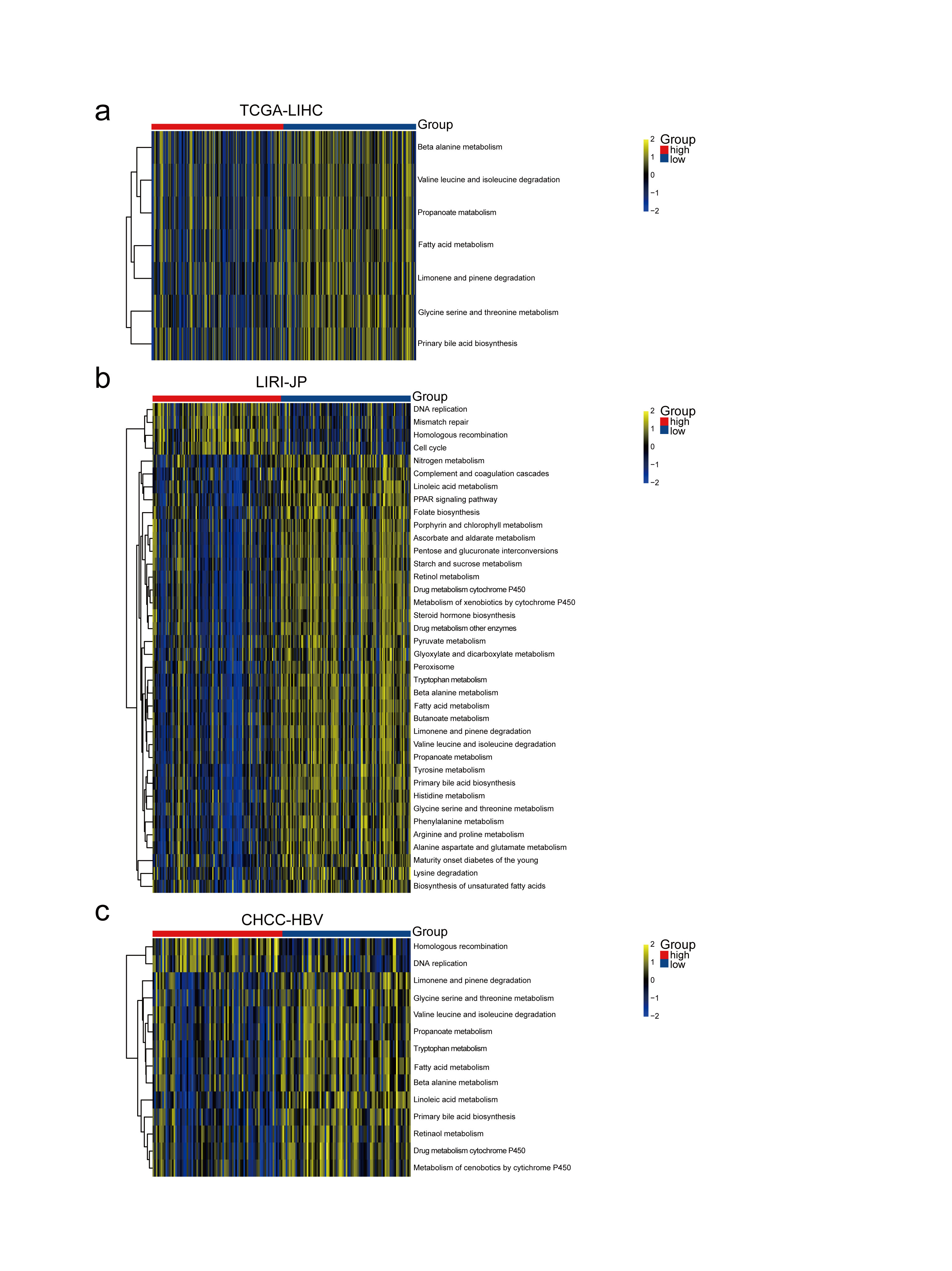

Supplement: Supplementary file 5 — Figure S5. Functional annotations of low‐ and high‐PSRS group. Heatmap illustrated the enrichment scores of differentially enriched molecular pathways evaluated by GSVA analysis between low‐ and high‐PSRS group in the TGCA‐LIHC (A), LIRI‐JP (B), and CHBB‐HBV (C). Yellow represented high enrichment scores, and blue represented low enrichment scores. [file CAM4-13-e70081-s002.tif]

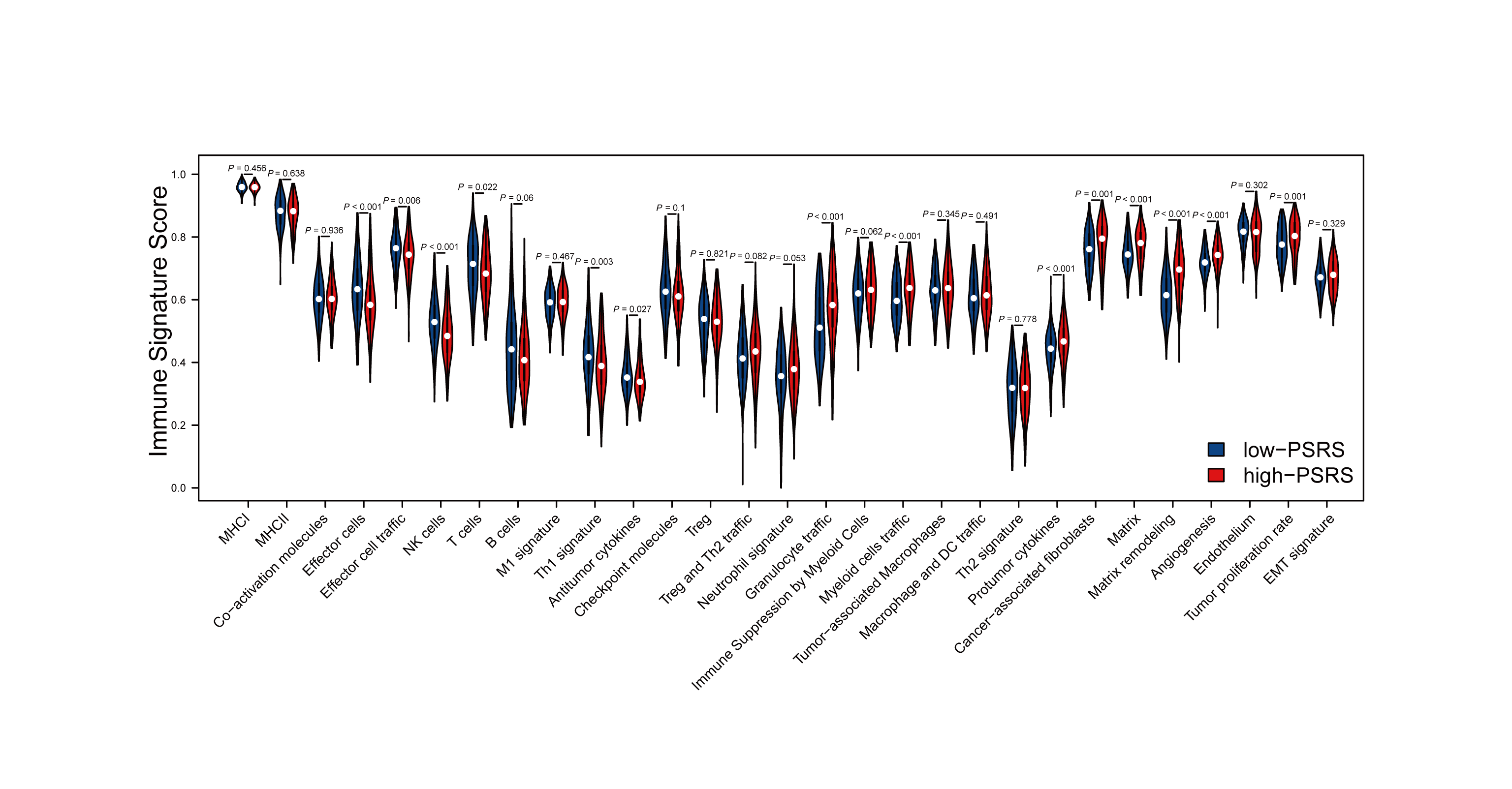

Supplement: Supplementary file 6 — Figure S6. Comparisons of the scores of 29 immune signatures in low‐ and high‐PSRS group. [file CAM4-13-e70081-s001.tif]

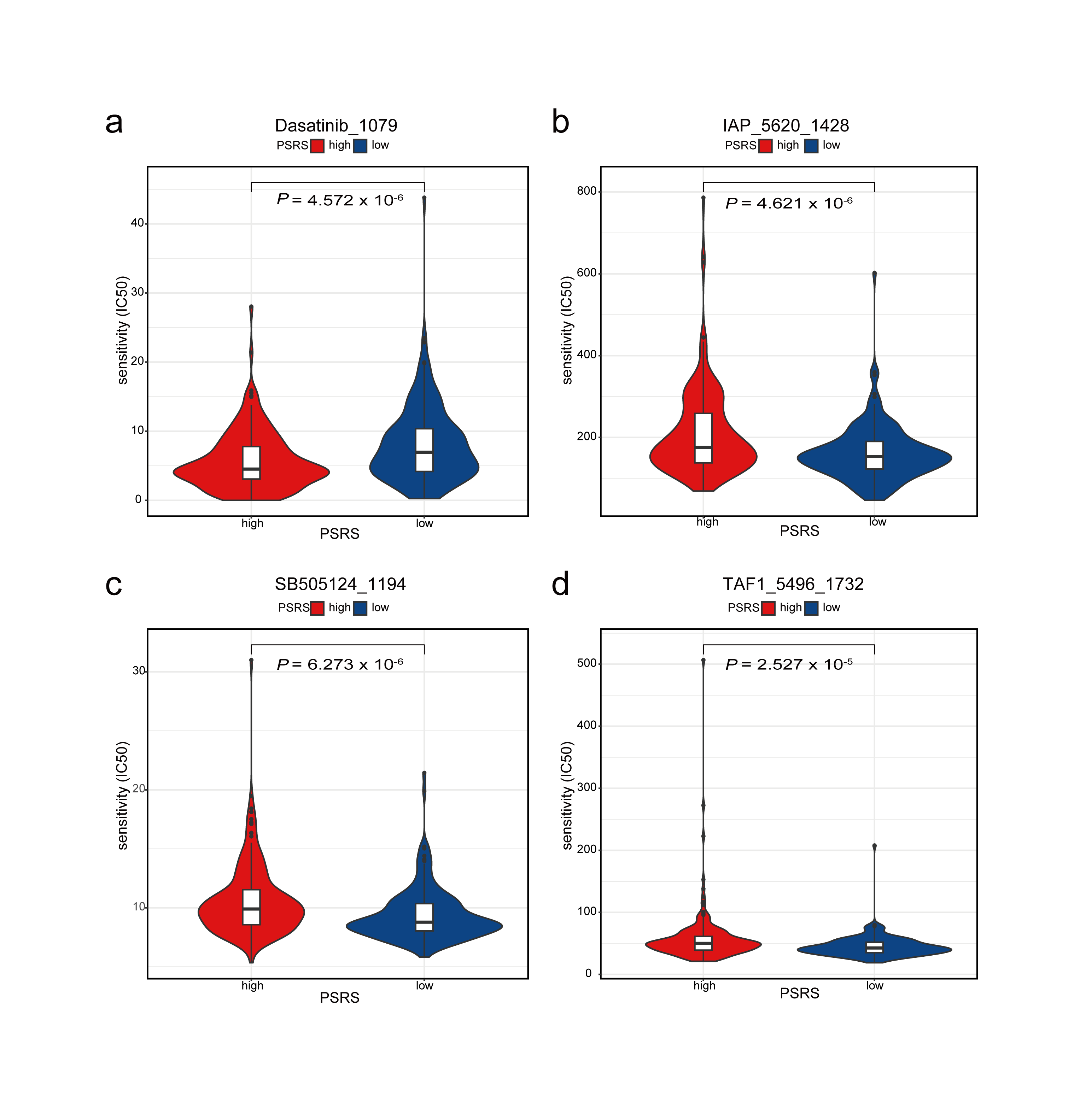

Supplement: Supplementary file 7 — Figure S7. The screened drugs for LIHC treatment. IC 50 value of Dasatinib_1079 (A), IAP_5620_1428 (B), SB505124_1194 (C), and TAF1_5496_1732 (D) in high‐and low‐PSRS patients with LIHC. [file CAM4-13-e70081-s006.tif]
