# Supplementary material for: Identification of pyroptosis subtypes and prognosis model of hepatocellular carcinoma based on pyroptosis‐related genes
Source: Cancer Med. 2024 Aug 9;13(15):e70081. doi: 10.1002/cam4.70081 (PMC11316015; doi:10.1002/cam4.70081)
Supplement: Supplementary file 8 — Table S1. Pyroptosis‐related genes. [file CAM4-13-e70081-s008.doc]

**Table S1.** Pyroptosis related genes.

| **Genes** | **Full-names** |
| --- | --- |
| ***AIM2*** | Absent in melanoma 2 |
| ***APIP*** | APAF1 interacting protein |
| ***BAK1*** | BCL2 antagonist/killer 1 |
| ***BAX*** | BCL2 associated X, apoptosis regulator |
| ***CASP1*** | Caspase 1 |
| ***CASP3*** | Caspase 3 |
| ***CASP4*** | Caspase 4 |
| ***CASP5*** | Caspase 5 |
| ***CASP6*** | Caspase 6 |
| ***CASP8*** | Caspase 8 |
| ***CASP9*** | Caspase 9 |
| ***CHMP2A*** | Charged multivesicular body protein 2A |
| ***CHMP2B*** | Charged multivesicular body protein 2B |
| ***CHMP3*** | Charged multivesicular body protein 3 |
| ***CHMP4A*** | Charged multivesicular body protein 4A |
| ***CHMP4B*** | Charged multivesicular body protein 4B |
| ***CHMP6*** | Charged multivesicular body protein 6 |
| ***CHMP7*** | Charged multivesicular body protein 7 |
| ***CYCS*** | Cytochrome c, somatic |
| ***DHX9*** | DExH-box helicase 9 |
| ***DPP9*** | Dipeptidyl peptidase 9 |
| ***ELANE*** | Elastase, neutrophil expressed |
| ***GPX4*** | Glutathione peroxidase 4 |
| ***GSDMA*** | Gasdermin A |
| ***GSDMB*** | Gasdermin B |
| ***GSDMC*** | Gasdermin C |
| ***GSDMD*** | Gasdermin D |
| ***GSDME*** | Gasdermin E |
| ***GZMA*** | Granzyme A |
| ***GZMB*** | Granzyme A |
| ***HMGB1*** | High mobility group box 1 |
| ***IL18*** | Interleukin 18 |
| ***IL1A*** | Interleukin 1 alpha |
| ***IL1B*** | Interleukin 1 beta |
| ***IL6*** | Interleukin 6 |
| ***IRF1*** | Interferon regulatory factor 1 |
| ***IRF2*** | Interferon regulatory factor 2 |
| ***MAP3K20*** | Mitogen-activated protein kinase kinase kinase 20 |
| ***MEFV*** | MEFV innate immuity regulator, pyrin |
| ***NAIP*** | NLR family apoptosis inhibitory protein |
| ***NLRC4*** | NLR family CARD domain containing 4 |
| ***NLRP1*** | NLR family pyrin domain containing 1 |
| ***NLRP2*** | NLR family pyrin domain containing 2 |
| ***NLRP3*** | NLR family pyrin domain containing 3 |
| ***NLRP6*** | NLR family pyrin domain containing 6 |
| ***NLRP7*** | NLR family pyrin domain containing 7 |
| ***NLRP9*** | NLR family pyrin domain containing 9 |
| ***NOD1*** | Nucleotide binding oligomerization domain containing 1 |
| ***NOD2*** | Nucleotide binding oligomerization domain containing 2 |
| ***PJVK*** | Pejvakin |
| ***PLCG1*** | Phospholipase C gamma 1 |
| ***PRKACA*** | Protein kinase cAMP-activated catalytic subunit alpha |
| ***PYCARD*** | PYD and CARD domain containing |
| ***SCAF11*** | SR-related CTD associated factor 11 |
| ***TIRAP*** | TIR domain containing adaptor protein |
| ***TNF*** | Tumor necrosis factor |
| ***TP53*** | Tumor protein p53 |
| ***TP63*** | Tumor protein p63 |
| ***TREM2*** | Triggering receptor expressed on myeloid cells 2 |
| ***ZBP1*** | Z-DNA binding protein 1 |
