# Supplementary material for: Identification of pyroptosis subtypes and prognosis model of hepatocellular carcinoma based on pyroptosis‐related genes
Source: Cancer Med. 2024 Aug 9;13(15):e70081. doi: 10.1002/cam4.70081 (PMC11316015; doi:10.1002/cam4.70081)
Supplement: Supplementary file 9 — Table S2. Small molecular compounds with significantly different responses between high‐PSRS and low‐PSRS groups. [file CAM4-13-e70081-s004.doc]

**Table S2.** Small molecular compounds with significantly different responses between high-PSRS and low-PSRS groups.

| **Drugs** | **Pvalue** |
| --- | --- |
| **Dasatinib_1079** | 4.572 x 10^-6^ |
| IAP_5620_1428 | 4.621 x 10^-6^ |
| SB505124_1194 | 6.273 x 10^-6^ |
| TAF1_5496_1732 | 2.527 x 10^-5^ |
| OF-1_1853 | 6.179 x 10^-5^ |
| Staurosporine_1034 | 1.253 x 10^-4^ |
| LCL161_1557 | 2.314 x 10^-4^ |
| Doramapimod_1042 | 2.527 x 10^-4^ |
| ERK_6604_1714 | 2.795 x 10^-4^ |
| MK-1775_1179 | 3.802 x 10^-4^ |
| Afatinib_1032 | 6.628 x 10^-4^ |
| Lapatinib_1558 | 7.257 x 10^-4^ |
| YK-4-279_1239 | 7.560 x 10^-4^ |
| AZD7762_1022 | 9.879 x 10^-4^ |
| PRT062607_1631 | 1.706 x 10^-3^ |
| Vinblastine_1004 | 1.787 x 10^-3^ |
| AZD6738_1917 | 1.842 x 10^-3^ |
| Ulixertinib_2047 | 1.892 x 10^-3^ |
| GDC0810_1925 | 2.330 x 10^-3^ |
| BPD-00008900_1998 | 2.374 x 10^-3^ |
| Ulixertinib_1908 | 2.577 x 10^-3^ |
| AZD6482_2169 | 2.796 x 10^-3^ |
| Osimertinib_1919 | 2.869 x 10^-3^ |
| Wee1 Inhibitor_1046 | 3.065 x 10^-3^ |
| JAK1_8709_1718 | 3.470 x 10^-3^ |
| BDP-00009066_1866 | 3.663 x 10^-3^ |
| Dihydrorotenone_1827 | 4.303 x 10^-3^ |
| VX-11e_2096 | 4.458 x 10^-3^ |
| Uprosertib_2106 | 4.522 x 10^-3^ |
| SCH772984_1564 | 4.921 x 10^-3^ |
| Trametinib_1372 | 5.222 x 10^-3^ |
| WIKI4_1940 | 5.407 x 10^-3^ |
| Cediranib_1922 | 5.426 x 10^-3^ |
| Paclitaxel_1080 | 5.696 x 10^-3^ |
| ULK1_4989_1733 | 6.102 x 10^-3^ |
| 5-Fluorouracil_1073 | 9.259 x 10^-3^ |
| Dinaciclib_1180 | 9.290 x 10^-3^ |
| ML323_1629 | 9.351 x 10^-3^ |
| Vinorelbine_2048 | 9.919 x 10^-3^ |
| Docetaxel_1819 | 1.035 x 10^-2^ |
| Telomerase Inhibitor IX_1930 | 1.048 x 10^-2^ |
| Cyclophosphamide_1512 | 1.231 x 10^-2^ |
| MG-132_1862 | 1.304 x 10^-2^ |
| AZD1208_1449 | 1.320 x 10^-2^ |
| Vincristine_1818 | 1.456 x 10^-2^ |
| Tozasertib_1096 | 1.488 x 10^-2^ |
| Axitinib_1021 | 1.598 x 10^-2^ |
| Sapitinib_1549 | 1.674 x 10^-2^ |
| Buparlisib_1873 | 1.679 x 10^-2^ |
| VE821_2111 | 1.700 x 10^-2^ |
| I-BRD9_1928 | 1.710 x 10^-2^ |
| Docetaxel_1007 | 1.715 x 10^-2^ |
| EPZ5676_1563 | 1.758 x 10^-2^ |
| Temozolomide_1375 | 1.763 x 10^-2^ |
| Nilotinib_1013 | 1.874 x 10^-2^ |
| BMS-536924_1091 | 2.184 x 10^-2^ |
| Sepantronium bromide_1941 | 2.538 x 10^-2^ |
| BMS-754807_2171 | 2.575 x 10^-2^ |
| Mitoxantrone_1810 | 3.174 x 10^-2^ |
| Sabutoclax_1849 | 3.174 x 10^-2^ |
| PAK_5339_1730 | 3.238 x 10^-2^ |
| AZD5991_1720 | 3.712 x 10^-2^ |
| Bortezomib_1191 | 3.998 x 10^-2^ |
| Acetalax_1804 | 4.455 x 10^-2^ |
| Mirin_1048 | 4.700 x 10^-2^ |
